# Zinc defends against Parthanatos and promotes functional recovery after spinal cord injury through SIRT3‐mediated anti‐oxidative stress and mitophagy

**DOI:** 10.1111/cns.14222

**Published:** 2023-04-17

**Authors:** Dingyuan Jiang, Xu Yang, Minghao Ge, Hengshuo Hu, Chang Xu, Shan Wen, Hao Deng, Xifan Mei

**Affiliations:** ^1^ Suzhou Medical College of Soochow University Suzhou China; ^2^ Department of Spinal Surgery Zhuzhou 331 Hospital Zhuzhou China; ^3^ Department of Orthopedics The First Affiliated Hospital of Jinzhou Medical University Jinzhou China; ^4^ Department of Orthopedics The Third Affiliated Hospital of Jinzhou Medical University Jinzhou China; ^5^ Key Laboratory of Tissue Engineering of Liaoning Province Jinzhou Medical University Jinzhou China

**Keywords:** anti‐oxidative stress, mitophagy, Parthanatos, SIRT3, spinal cord injury, zinc

## Abstract

**Introduction:**

Spinal cord injury (SCI) is a central nervous system injury that is primarily traumatic and manifests as motor, sensory, and autonomic dysfunction below the level of damage. Our previous studies confirmed the ability of zinc to protect mitochondria, protect neurons and promote spinal cord recovery. However, the role of zinc in Parthanatos is unknown.

**Aim:**

We investigated the effects of zinc in Parthanatos from oxidative stress and mitophagy. We elucidated the role of SIRT3 in providing new ideas for treating spinal cord injury.

**The Results:**

Zinc protected SCI mice by regulating Parthanatos. On the one hand, zinc eliminated ROS directly through SIRT3 deacetylation targeting SOD2 to alleviate Parthanatos. On the other hand, zinc eliminated ROS indirectly through SIRT3‐mediated promotion of mitophagy to alleviate Parthanatos.

**Conclusion:**

Zinc defends against Parthanatos and promotes functional recovery after spinal cord injury through SIRT3‐mediated anti‐oxidative stress and mitophagy.

## Graphical abstract

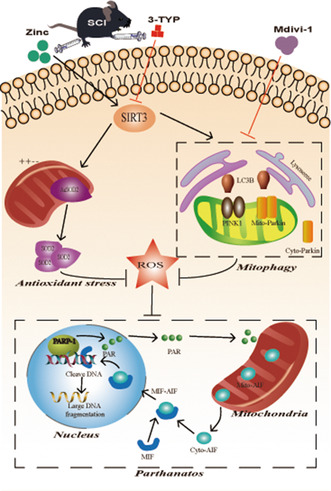

## 1 INTRODUCTION

Spinal cord injury (SCI) is a central nervous system injury that is primarily traumatic and manifests as motor, sensory, and autonomic dysfunction below the level of injury.^1^ Decreased quality of life and complications for patients with spinal cord injuries impose a severe economic burden on families and society.^2^ There is no satisfactory clinical treatment for spinal cord injury, mainly because of the complexity of its pathophysiology.^3^, ^4^ The pathophysiology of the spinal cord after the injury is divided into two stages: primary and secondary. Prior injury is mechanical damage resulting in substantial destruction of neurons. Secondary injury mainly manifests by ischemia and hypoxia, vasogenic edema, mitochondrial dysfunction, and excessive reactive oxygen species (ROS) production. Among these mechanisms, excessive production of ROS plays a key role, further damaging neuronal components such as nucleic acids, proteins, and lipids and inflammation and activation of various programmed cell death pathways in neurons.^5^

Parthanatos is a poly ADP‐ribose polymerase‐1 (PARP‐1) ‐dependent programmed death.^6^, ^7^ That is important in neurological diseases such as neurodegenerative diseases, cerebrovascular diseases, spinal cord injuries, and gliomas.^8^ Mechanistically, the DNA damage sensor PARP‐1 is hyper activated and produces large amounts of poly ADP‐ribose (PAR) released into the cytoplasm, inducing depolarization of mitochondria and promoting the release of apoptosis‐inducing factor (AIF) from mitochondria into the cytoplasm.^9^, ^10^ AIF binds to macrophage migration inhibitory factor (MIF) in the cytoplasm and enters the nucleus, causing chromatin and DNA breaks, thereby accelerating cell death.^8^, ^11^ Also, extensive studies have shown that pathological changes after spinal cord injury eventually lead to accumulating reactive oxygen species (ROS), which can damage DNA and activate Parthanatos.^12^

Mitochondria play an essential role in cellular activity as the leading site for maintaining cellular energy metabolism and mitochondrial homeostasis.^13^ Increasing evidence suggests that the secondary injury phase after spinal cord injury is closely associated with mitochondrial damage and ROS accumulation.^14^, ^15^, ^16^ Mitophagy, selective autophagic degradation of damaged mitochondria,^17^ can reduce ROS accumulation.^18^, ^19^ SIRT3 is a member of the Sirtuins family of proteins localized in mitochondria and characterized as a deacetylase that deacetylates associated with acetylated proteins in mitochondria.^20^ In addition, it has been shown that SIRT3 is involved in maintaining mitochondrial function and regulating ROS.^20^, ^21^

A promising strategy is to intervene with Parthanatos to treat spinal cord injury by protecting mitochondria, scavenging reactive oxygen species, or reducing the accumulation of reactive substances.

Zinc is essential for vital activity and binds with numerous proteins (enzymes and transcription factors).^22^, ^23^ Approximately 10% of human proteins are zinc proteins, and most human zinc‐binding proteins regulate gene expression.^24^ Previous literature suggests that Sirtuin catalyzes the characteristic sequence motifs corresponding to the core structural domain, including the zinc‐binding site.^25^, ^26^, ^27^ This feature determines that zinc affects almost all aspects of cell biology and is essential for maintaining cellular homeostasis.^28^ Studies have shown that zinc is involved in oxidative stress, inflammatory response, and immune regulation.^23^, ^29^, ^30^ Our previous studies confirmed the ability of zinc to protect mitochondria, protect neurons and promote spinal cord recovery.^31^, ^32^ However, the role of zinc in Parthanatos is unknown.

In this study, we investigated the effects of zinc in Parthanatos from oxidative stress and mitophagy. We elucidated the role of SIRT3 in providing new ideas for treating spinal cord injury.

## 2 MATERIALS AND METHODS

### 2.1 Animal and drug treatment

All 8‐week‐old C57BL/6J female mice weighing 20–25 g (Liaoning Changsheng Biotechnology Co., Ltd.) were found in the SPF mouse house at Jinzhou Medical University. A 12‐h light/dark cycle was administered at 22 2°C, with adequate food and water available for mice. The Animal Protection and Use Committee of Jinzhou Medical University endorsed all experimental procedures used in this study.

The animals were randomly assigned to the Sham, SCI, ZnG, and 3‐TYP groups. Mice were extensively anesthetized by intrapulmonary injection with 1% sodium pentobarbital (50 mg/kg, P‐010, Sigma‐Aldrich). Mice were deeply anesthetized by intrapulmonary injection with 1% sodium pentobarbital (50 mg/kg, P‐010, Sigma‐Aldrich). Mice were profoundly anesthetized by intrapulmonary injection with 1% sodium pentobarbital (50 mg/kg, P‐010, Sigma‐Aldrich). Mice were deeply anesthetized by intrapulmonary injection with 1% sodium pentobarbital (50 mg/kg, P‐010, Sigma‐Aldrich). Mice were profoundly anesthetized by intrapulmonary injection with 1% sodium pentobarbital (50 mg/kg, P‐010, Sigma‐Aldrich).^33^ Partial laminectomy was performed on the T9/T10 vertebral body to expose the T9/T10 segment. An altered impactor (diameter: 2 mm. Weight: 12.5 g. Height: 1.5 cm) was used to cause a spinal cord contusion. The ZnG group was given an intraperitoneal injection of 30 mg/kg ZnG 2 h postoperatively, 1 time/day until day 3. The 3‐TYP group was assigned 50 mg/kg 3‐TYP intraperitoneally 1 week before surgery, 1 day every other day, three times,^33^ and postoperative treatment was the same as the ZnG group. The SCI group was given an intraperitoneal isotonic glucose injection 2 h postoperatively, 1 time/day until day 3. Only the laminae were removed in the Sham group, and postoperative isotonic glucose injection was administered intraperitoneally.

### 2.2 Behavioral assessment

The BMS score was used to assess motor function in the hind limbs of mice.^34^ Professionally trained operators were unaware of the experimental conditions and repeated the evaluation of the mice on three occasions. These mice were evaluated 1 h before surgery, and 1, 3, 7, 14, 21 and 28 days after surgery.

### 2.3 Ultrasonic imaging test

The urinary bladder volumes of mice in each group were measured at 28 days after surgery using an ultrasound diagnostic system (Ex. No. 9362, ESAOTE S.p.A Esaote Group) and a color Doppler ultrasound diagnostic instrument (Model No. DC‐6EII, Shenzhen Myriad Biomedical Electronics Co., Ltd.), and the volume calculations were performed on the ultrasound images.^35^ Specifically, mice were anesthetized using the intrapulmonary anesthesia injection with 1% sodium pentobarbital (50 mg/kg, P‐010, Sigma‐Aldrich). After fixing the mice in the prone position, the abdomen was first abdominally shaved, then cleaned with 70% ethanol and soap. A medical ultrasound coupling agent was applied to facilitate the propagation from the transducer to the skin. While maintaining slight pressure, the transducer was placed to visualize and digitally capture the maximum cross‐sectional area of the bladder, sequentially capturing the length, depth, and width, with the hypertonic bladder appearing as a black oval structure with the hyperbolic surrounding tissue. Ultrasound imaging system software automatically calculates the bladder volume.

### 2.4 Testing tissue preparation

At the designed time, mice were anesthetized and sequentially perfused with 0.9% NaCl and 4% paraformaldehyde for cardiac perfusion. A 0.5 cm spinal cord specimen was taken and fixed in 4% PFA for 48 h and then dehydrated sequentially using 4% paraformaldehyde solution containing 10%, 20%, and 30% sucrose. The area of the spinal cord 3 mm above and below the point of injury was cut with a frozen cut section to make frozen sections of 10 μm, stored at −80°C. The tissue was fixed for 48 h, dehydrated in gradient, soaked in xylene, and then cut into 7 μm paraffin sections after paraffin embedding and stored at room temperature.

### 2.5 Hematoxylin–eosin staining

Hematoxylin–eosin staining was performed using a commercial kit (#G1120 Solarbio), and paraffin sections were dewaxed, rehydrated, stained, dehydrated, transparent, and sealed sequences according to the instructions.

### 2.6 QRT‐PCR

After the euthanasia of mice, 1.5 cm of spinal cord tissue was extracted with the TRIzol reagent (Ambion) for total RNA. CDNA (Promega) was synthesized by reverse transcription with 5 μg of total RNA, followed by QRT‐PCR using SYBR Green (Promega) for QRT‐PCR. Amplification conditions were 2 min reaction at 95°C, 15 s reaction at 95°C, and 1 min reaction at 60°C for 40 cycles. The GAPDH control gene has been normalized at the target gene expression level. The corresponding target gene expression of the experimental group was compared with the control group using the (1 + e)ΔΔ*C*_T_ method. The primers (Beijing Dingguo Changsheng Biotechnology Co., Ltd.) used in the study are listed above in Table 1 (*n* = 3 mice/group).

**TABLE 1:** Primer sequences used in qRT‐PCR.

Gene	Primer sequence (5′–3′)	Product size (Bp)
SIRT3	Forward primer: TCTACAGCAACCTTCAGCAGTA	22
	Reverse primer: CAGGAAGTAGTGAGTGACATTGG	23
GAPDH	Forward primer: AATGGTGAAGGTGGTGTGA	20
	Reverse primer: CGCTCCTGGAAGATGGTGAT	20

### 2.7 Cell culture and viability assay

VSC4.1 cells were cultured with DMEM (Gibco) containing 10% fetal bovine serum and 0.4% penicillin–streptomycin at 37°C and 5% CO_2_. Cell drug toxicity tests were tested with the CCK‐8 kit (#CA1210, Solarbio). The cells were inoculated into 96‐well plates at 5000 cell/well density and incubated at 37°C in a CO_2_ incubator for 24 h. The cells were processed with various concentrations of ZnG, H_2_O_2_, Mdivi‐1, and 3‐TYP and placed at other times, followed by incubation with 10 μ CCK8 per well for 4 h. Finally, absorbance (DO) was measured at 450 nm with an enzymatic marker.

### 2.8 Cell treatment

The experiments were divided into Vehicle group, H_2_O_2_ group, ZnG group, Mdivi‐1 group, or 3‐TYP group. To simulate SCI oxidative stress, VSC.4.1 was treated with H_2_O_2_.^36^, ^37^ H_2_O_2_ treated VSC4.1 with H_2_O_2_ (80 μmol/L) for 3 h. The ZnG group was treated with H_2_O_2_ (80 μmol/L) for 3 h, followed by ZnG (100 μmol/L, # Z820656‐100 g, Macklin) for 24 h. The Mdivi‐1 group was treated with the mitophagy inhibitor Mdivi‐1 (25 μmol/L, # ab144589, Abcam) for 3 h before the treatment with the addition of H_2_O_2_, and the rest of the treatment was the same as the ZnG group.^38^ The 3‐TYP group was treated with the SIRT3 inhibitor 3‐TYP (50 μmol/L, # IT1960, Solarbio) before adding H_2_O_2_ for 3 h, and the rest of the treatment was the same as that of the ZnG group.^36^ The Vehicle group was cultured in DMEM, and other treatment factors were identical.

### 2.9 Western blotting

We used the same method as before to perform Western blotting.^37^ Before Western blotting, we used a total protein extraction kit (BC3710, Solarbio), cellular mitochondrial isolation kit (C3601, Beyotime) or tissue mitochondrial isolation kit (C3606, Beyotime), and nuclear protein extraction kit (R0050, Solarbio) to extract total protein, mitochondrial protein or de‐mitochondrial cytoplasmic protein, and nuclear protein, respectively. The primary antibodies used were anti‐PARP‐1 (1:1000, DF7198, Affinity); anti‐AIF (1:1000, BF0591, Affinity); anti‐COX IV (1:1000, AF5468, Affinity); anti‐Histone H3 (1:1000, AF0863, Affinity); anti‐β‐actin (1:10,000, 66009‐1‐Ig, Protentech); anti‐LC3BI/II (1:1000, A5402, Affinity); anti‐PINK1 (1:200, PA5‐85930, Invitrogen); anti‐Parkin (1:200, 702785, Invitrogen); anti‐SIRT3 (1:1000, Affinity, AF5135); anti‐SOD2/MnSOD (acetyl K68) (1:5000, ab137037, Abcam); and anti‐SOD2/MnSOD (1:5000, ab13533, Abcam). The second antibodies used were HRP‐conjugated AffiniPure Goat Anti‐Mouse IgG (1:10,000, Proteintech) and HRP‐conjugated AffiniPure Goat Anti‐Rabbit IgG (1:10,000, Proteintech). Finally, PVDF molds were visualized on a Tanon 2500R gel imaging system (Tanon) using a developer (Tanon), and the band intensity was quantified using ImageJ 1.39V software.

### 2.10 Immunofluorescent staining

VSC4.1 neurons or spinal cord frozen sections were fixed using 4% paraformaldehyde, then incubated with 0.3% Triton X‐100 for 10 min, bovine serum albumin (BSA) for 2 h, then incubated with primary antibody overnight at 4°C and with secondary antibody at room temperature for 2 h. In the end, the sections were blocked by incubating with DAPI at room temperature for 10 min. The primary antibodies are as follows: anti‐SIRT3 (1:500, AF5135, Affinity); anti‐PARP‐1 (1:500, DF7198, Affinity); anti‐AIF (1:500, BF0591, Affinity); anti‐NeuN (1:1000, ab104224, Abcam); anti‐β‐tubulin (1:500, 66240‐1‐Ig, Proteintech) The second antibodies are as follows: Alexa Fluor 488/568 goat anti‐mouse IgG, Alexa Fluor 568 goat anti‐rabbit IgG (1:1000, Thermo Fisher Scientific).

### 2.11 Transmission electron microscopy

Transmission electron microscopy (TEM) was used to monitor mitochondrial injury in the spinal cord, as described previously.^39^

### 2.12 Mitochondrial membrane potential detection

Mitochondrial membrane potential was detected using the JC‐1 kit (#C2006, Beyotime Institute of Biotechnology). The fluorescence intensity of each group of cells was observed directly using a fluorescence microscope after staining VSC4.1 cells. After fresh spinal cord tissue was taken from mice, mitochondria were extracted using the Tissue Mitochondrial Isolation Kit (#C3606, Beyotime), then incubated with a JC‐1 working solution for 20 min, and the fluorescence intensity was detected using an enzyme‐labeled fluorometer.

### 2.13 Reactive oxygen detection

Cells were inoculated in 24‐well plates and treated accordingly. Cells were incubated with DCFH‐DA Reactive Oxygen Species ROS Fluorescent Probe (#D6470, Solarbio) at 37°C for 20 min and then by fluorescence microscopy. Fresh spinal cord tissue homogenates were taken from each group of mice and assayed by a mouse (Mouse) reactive oxygen species (ROS) ELISA kit (#JK652393, Shanghai Enzyme Link Biological Co., Ltd.). Finally, the absorbance (OD) was measured at 450 nm wavelength with an enzyme marker to calculate the sample activity.

### 2.14 8‐OH‐dG detection

The 8‐OH‐dG content of each mouse spinal cord tissue group and VSC 4.1 cells was measured by ELISA assay kit (#YX‐E20120, Wuhan Yipu Biotechnology Co., Ltd.), respectively. The absorbance (OD value) was measured at 450 nm wavelength using an enzyme marker, and the sample concentrations were calculated.

### 2.15 Determination of manganese superoxide dismutase (Mn‐SOD) enzyme activity

Mn‐SOD activity assay kit (#S0103, Beyotime Institute of Biotechnology) was used to detect Mn‐SOD enzyme activity in spinal cord tissue and VSC4.1 cells of each group of mice, respectively.

### 2.16 Mitophagy detection

A mitophagy assay kit (MD01, Dongren Chemical Technology (Shanghai) Co., Ltd.) was used for the assay. Specifically, first, cells were inoculated in confocal dishes for 24–48 h to remove the medium, washed twice using serum‐free medium, and then added an appropriate volume of 10 mmol/L Mitophagy Dye working solution and incubated at 37°C for 30 min; Second, each group was treated with the proper treatment and then added 1 μmol/L Lyso Dye working solution and incubated at 37°C for 30 min; finally, the groups were washed two more times with serum‐free medium and observed under the fluorescence microscope.

### 2.17 Statistical analysis

Data were analyzed using SPSS Software, version 25.0. The Shapiro–Wilk test was used to assess the data distribution. Normally distribution date is expressed as mean ± standard deviation. In cases with more than two groups, we used a one‐way analysis of variance (ANOVA) followed by Bonferroni's post hoc test when the variance was equal and a Games–Howell post hoc test when the variance was uneven. The non‐parametric Kruskal–Wallis *H* test analyzed Basso Mouse Scale (BMS) scores. Statistics are deemed significant when *p < 0.05. *p < 0.05, **p < 0.01 and ***p < 0.001.

## 3 RESULTS

### 3.1 Zinc can inhibit spinal cord neurons' Parthanatos after SCI

**FIGURE 1:**
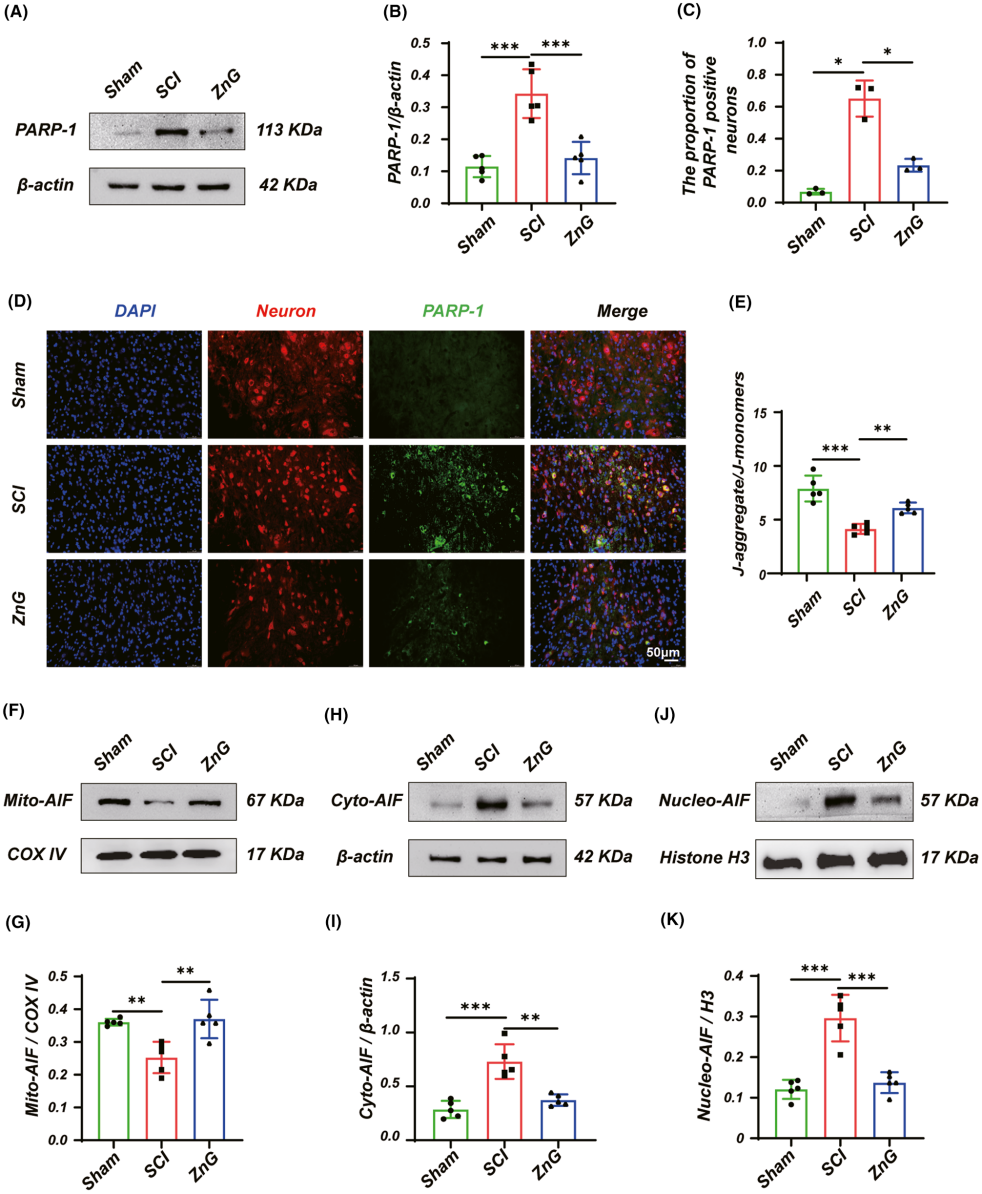
Zinc can inhibit Parthanatos in the spinal cord of SCI mice. (A) Western blot and (B) statistical results of western blots of PARP‐1 (*n* = 5, one‐way ANOVA followed by Bonferroni's post hoc test). (C) Immunofluorescence and (D) statistical analysis of immunofluorescence staining of PARP‐1 in nerve cells from each group (Scale bar = 50 μm, *n* = 3, one‐way ANOVA followed by Games–Howell post hoc test). (E) Mitochondrial membrane potential was measured with the JC‐1 probe (*n* = 5, one‐way ANOVA followed by Bonferroni's post hoc test). (F, H, J) Western blot and (G, I, K) statistical results of western blots of Mito‐AIF, Cyto‐AIF, and Nucleo‐AIF (*n* = 5, one‐way ANOVA followed by Bonferroni's post hoc test). The data represent means ± SD, * means, *p* < 0.05; ** means, *p* < 0.01; and *** means, *p* < 0.001.

To clarify whether PARP‐1‐dependent Parthanatos occurred after SCI. First, we examined the protein expression of PARP‐1 by western blotting and immunofluorescent staining, respectively. Western blotting results showed a significantly higher PARP‐1 expression in the SCI group than in the Sham group. At the same time, it decreased significantly in the ZnG group compared with the SCI group (Figure 1A,B). Similar results were obtained by immunofluorescence staining, indicating that ZnG could reduce the expression of PARP‐1 (Figure 1C,D). JC‐1 is an ideal fluorescent probe widely used to detect mitochondrial membrane potential in isolated mouse spinal cord tissue. Our results showed that the membrane potential was significantly reduced in the SCI group compared to Sham. At the same time, it was restored to the ZnG group, suggesting that mitochondrial depolarization occurred after SCI, while ZnG therapy significantly weakened mitochondrial depolarization (Figure 1E). Nuclear translocation of AIF was another feature of Parthanatos, with reduced Mito‐AIF expression and increased Cyto‐AIF and Nucleo‐AIF after SCI compared to the Sham group, and ZnG treatment suppressed these expressions (Figure 1F–K). Overall, activation of PARP‐1 after SCI decreased the membrane potential and accelerated nuclear translocation, features that are consistent with the manifestation of Parthanatos, confirming the occurrence of Parthanatos after SCI, which the administration of ZnG treatment significantly inhibited.

### 3.2 Zinc can promote mitophagy in spinal cord neurons after SCI

**FIGURE 2:**
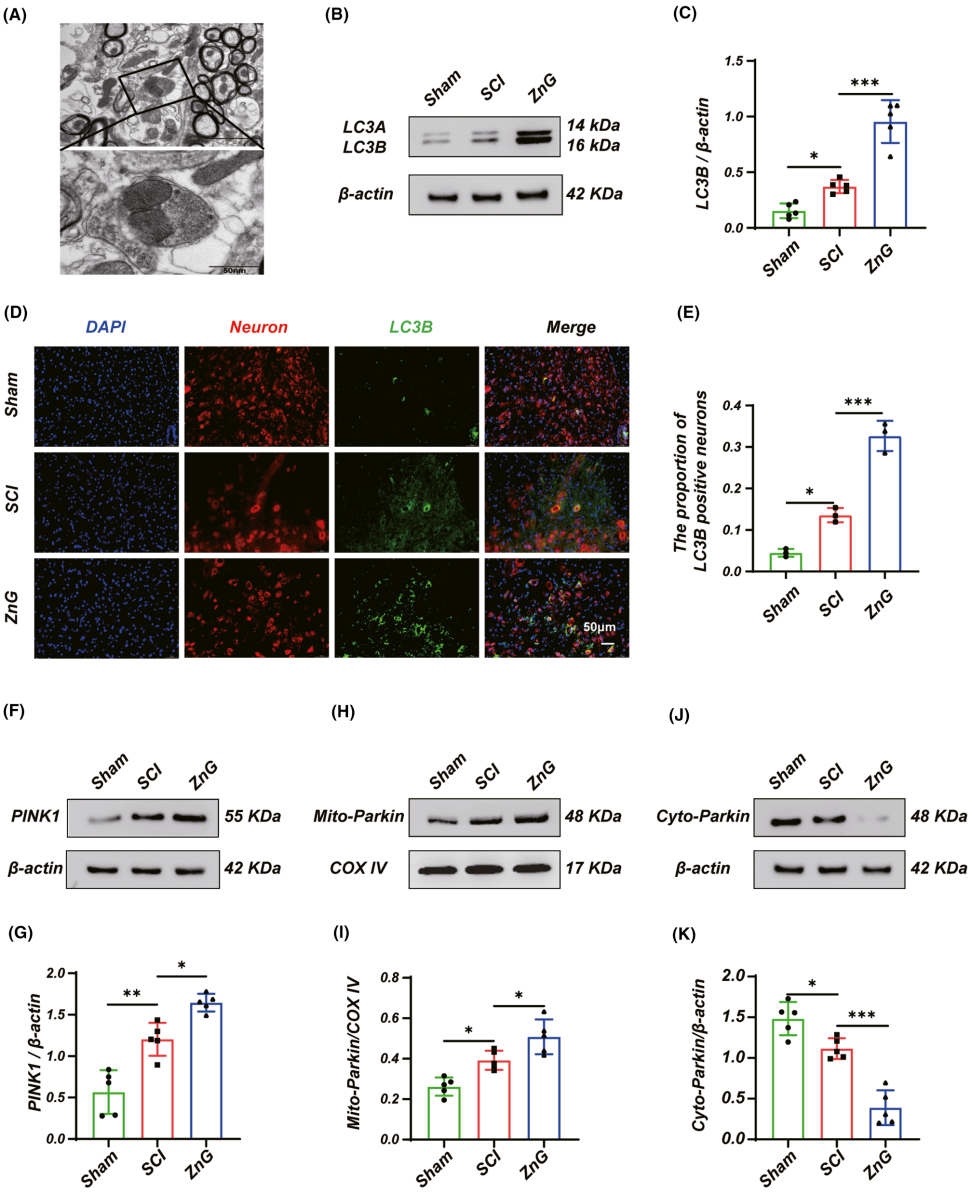
Zinc can promote mitophagy in the spinal cord of SCI mice. (A) Transmission electron microscopy reveals mitochondrial autophagosomes after administration of zinc treatment in spinal cord injured mice (*n* = 5, Scale bar = 2 μm, and 500 nm). (B) Western blot and (C) statistical results of western blots of LC3BI/II (*n* = 5, one‐way ANOVA followed by Bonferroni's post hoc test). (D) Immunofluorescence and (E) statistical analysis of immunofluorescence staining of LC3B in nerve cells from each group (Scale bar = 50 μm, *n* = 3, one‐way ANOVA followed by Bonferroni's post hoc test). (F, H, J) Western blot and (G, I, K) statistical results of western blots of PINK1, Mito‐Parkin, and Cyto‐Parkin (*n* = 5, one‐way ANOVA followed by Bonferroni's post hoc test). The data represent means ± SD, * means, *p* < 0.05; ** means, *p* < 0.01; and *** means, *p* < 0.001.

**FIGURE 3:**
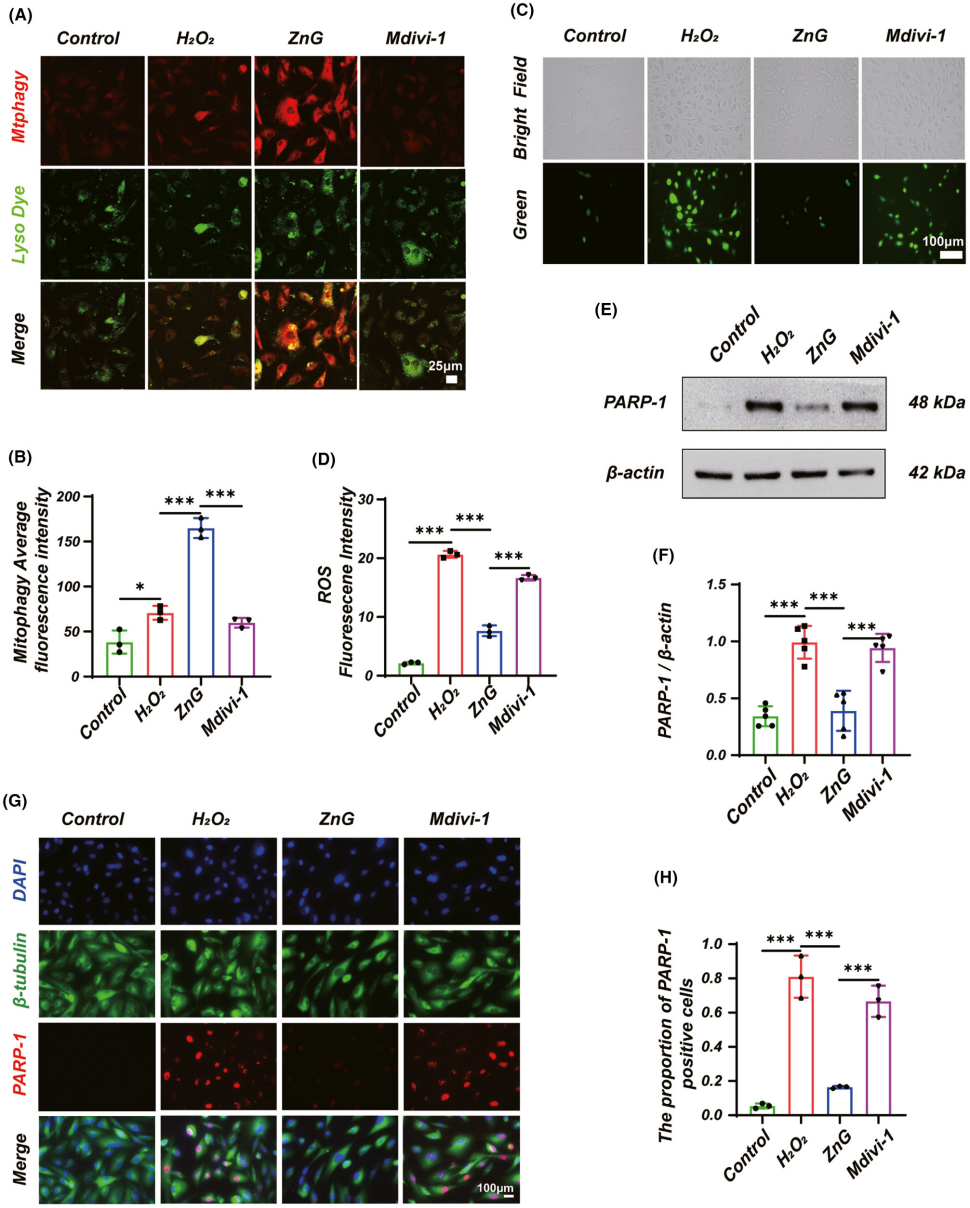
Zinc can reduce ROS accumulation through mitophagy, thus regulating PARP‐1‐dependent Parthanatos in VSC4.1 neurons. (A) The phenomenon of mitophagy was detected by the Mitophagy Detection Kit and (B) statistical results of red fluorescence intensity. (*N* = 3, Scale bar = 25 μm, all the data are expressed as means ± SD, one‐way ANOVA followed by Bonferroni's post hoc test). (C) ROS was measured by DCFH‐DA and (D) statistical results of fluorescence intensity. (*N* = 3, Scale bar = 100 μm, all the data are expressed as means ± SD, one‐way ANOVA followed by Bonferroni's post hoc test). (E) Western blot and (F) statistical results of western blots of PARP‐1 (*n* = 5, all the data are expressed as means ± SD, one‐way ANOVA followed by Bonferroni's post hoc test). (G) Immunofluorescence and (H) statistical analysis of immunofluorescence staining of PARP‐1 in nerve cells from each group (*n* = 3, Scale bar = 100 μm, one‐way ANOVA followed by Bonferroni's post hoc test). The data represent means ± SD, *p < 0.05, **p < 0.01, ***p < 0.001.

Mitophagy is a specific type of autophagy that maintains mitochondrial homeostasis. New evidence suggests that mitophagy protects neurons. Using transmission electron microscopy, we found broken mitochondria surrounded by bilayer vesicles after ZnG treatment (Figure 2A), indicating the presence of mitophagy after ZnG treatment. To further clarify the changes in mitophagy, we first detected changes in the mitophagy protein, and the level of LC3BI/II was significantly increased in the ZnG group (Figure 2B,C). We further detected immunofluorescence staining and obtained similar results (Figure 2D,E), indicating that zinc promotes mitophagy after SCI. In order to continue exploring the mechanism of mitophagy activation, we measured Parkin and PINK1 after SCI. Western blotting showed that ZnG treatment promoted activation of the PINK1/Parkin pathway and increased autophagic flux after SCI, as evidenced by an increase in PINK1, Mito‐Parkin, and Cyto‐Parkin was decreased (Figure 3F–K). In summary, zinc can promote Parkin and PINK1 pathways for mitophagy.

### 3.3 Inhibits mitophagy, increases the accumulation of ROS, and reverses the effect of zinc on Parthanatos

In vitro, to clarify whether zinc can regulate mitophagy, and secondly, to further verify the role of mitophagy in the protection of neurons by ZnG, we treated VSC.4.1 with H_2_O_2_ to simulate spinal cord neuronal injury and intervened with ZnG or/and Mdivi‐1, respectively. The CCK‐8 assay for H_2_O_2_ and ZnG determined the concentrations used to be 80 and 100 μmol/L, respectively (Figure S1A,B). We labeled VSC4.1 mitochondria using Mitophagy Dye, which produces a stronger red fluorescence when mitophagy occurs. The fluorescence intensity of Mitophagy Dye was significantly enhanced in the ZnG group compared to the H_2_O_2_ group and significantly weakened in the Mdivi‐1 group compared to the ZnG group (Figure 3A,B). In addition, we also used DCFH‐DA fluorescent probe to detect ROS and found that the fluorescence intensity of the Mdivi‐1 group was higher than that of the ZnG group (Figure 3C,D). In conclusion, zinc can promote H_2_O_2_‐induced mitophagy of VSC4.1 neurons and reduce ROS production, thus protecting neurons.

**FIGURE 4:**
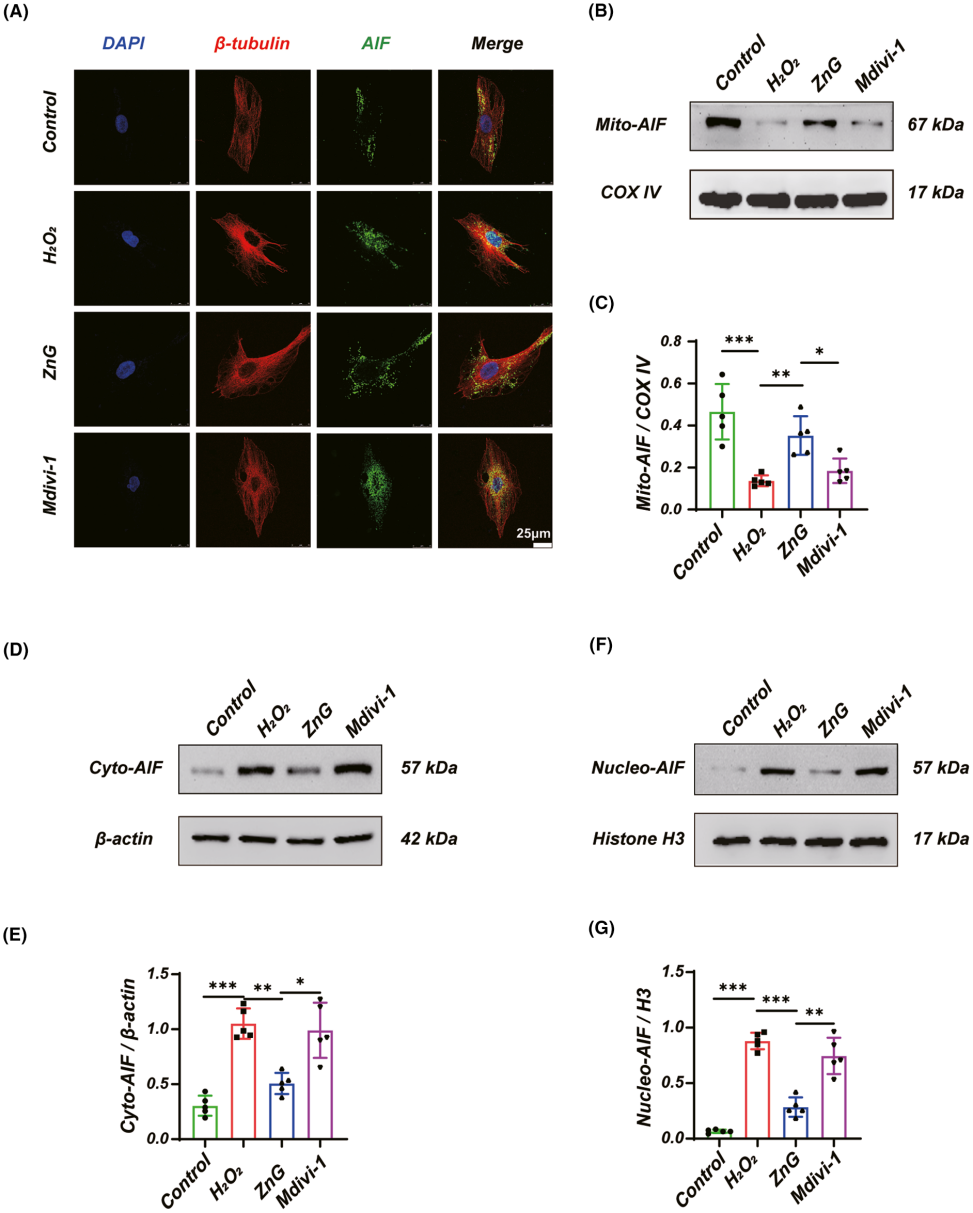
Inhibits mitophagy reverses the inhibitory effect of zinc on AIF nuclear translocation in VSC4.1 neurons. (A) Immunofluorescence staining was used to observe the nuclear translocation of AIF (*n* = 5, Scale bar = 25 μm). (B) Western blot and (C) statistical results of western blots of Mito‐AIF (*n* = 5, one‐way ANOVA followed by Bonferroni's post hoc test). (D, F) Western blot and (E, G) statistical results of western blots of Cyto‐AIF and Nucleo‐AIF (*n* = 5, one‐way ANOVA followed by Games‐Howell post hoc test). The data represent means ± SD, *p < 0.05, **p < 0.01, ***p < 0.001.

ROS is known as a trigger for Parthanatos as well. Therefore, we further demonstrated whether ZnG reduces ROS by promoting mitochondrial autophagy, thus alleviating the occurrence of Parthanatos. Western blotting suggested that PARP‐1 expression was decreased in VSC4.1 neurons after ZnG treatment, and pretreatment with Mdivi‐1 significantly reversed the treatment effect of ZnG (Figure 3E,F). We also used immunofluorescence to assess PARP‐1 expression and obtained similar results (Figure 3G,H). In addition, we also detected by immunofluorescence that AIF nuclear translocation was observed in the H_2_O_2_ group, ZnG reduced AIF nuclear translocation, and Mdivi‐1 reversed the denuclearization of ZnG in the Mdivi‐1 group (Figure 4A). We obtained similar results using Western blotting to detect changes in the AIF in mitochondria, cytoplasm, and nucleus, respectively (Figure 4B–G).

In summary, zinc can reduce Parthanatos by favoring mitophagy by reducing the production of ROS.

### 3.4 Zinc mediates anti‐oxidative stress via SIRT3

**FIGURE 5:**
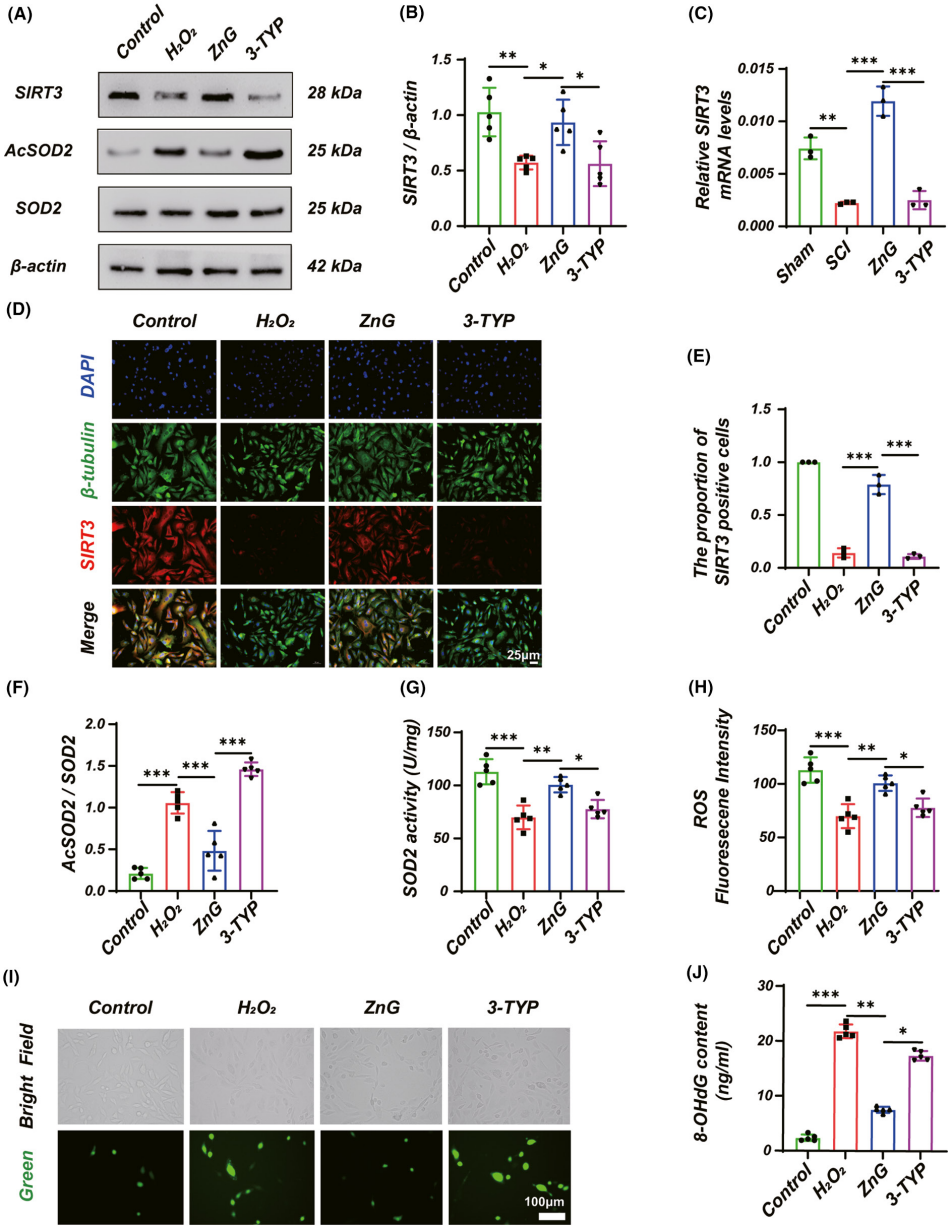
Zinc mediates anti‐oxidative stress via SIRT3. (A) Western blot and (B, F) statistical results of western blots of SRIT3 and AcSOD2/SOD2 (*n* = 5, one‐way ANOVA followed by Bonferroni's post hoc test). (C) Quantitative real‐time PCR of SIRT3 (*n* = 5, one‐way ANOVA followed by Bonferroni's post hoc test). (D) Immunofluorescence and (E) statistical analysis of immunofluorescence staining of SIRT3 from each group (*n* = 3, Scale bar = 25 μm, one‐way ANOVA followed by Bonferroni's post hoc test). (G) Statistical analysis of SOD2 activity (*n* = 5, one‐way ANOVA followed by Bonferroni's post hoc test). (I) ROS was measured by DCFH‐DA and (H) statistical results of fluorescence intensity (*n* = 5, Scale bar = 100 μm, one‐way ANOVA followed by Bonferroni's post hoc test). (J) 8‐OHdG content was detected by the 8‐OHdG assay kit (*n* = 5, one‐way ANOVA followed by Games–Howell post hoc test). The data represent means ± SD, *p < 0.05, **p < 0.01, ***p < 0.001.

It has been shown that SIRT3 can target SOD2 to regulate ROS, so we examined the changes of SIRT3 by Western blotting, QRT‐PCR, and immunofluorescence, respectively. In vitro, western blotting suggested increased expression of SIRT3 by ZnG treatment, while intervention and validation were performed using 3‐TYP, a specific inhibitor of SIRT3, which significantly reversed the therapeutic effect of ZnG. (Figure 5A,B). The same results were obtained for immunofluorescence assays (Figure 5D,E). We further performed QRT‐PCR experiments on mice, the mRNA level in the ZnG group was significantly increased compared with the SCI group, and the 3‐TYP group could dramatically reverse the promoting effect of ZnG (Figure 5C). We also performed the validation of SOD2 and ROS downstream of SIRT3. Previous studies have shown that SIRT3 regulates ROS through the deacetylation of SOD2. When we administered ZnG treatment after H_2_O_2_ simulated oxidative stress, AcSOD2/SOD2 ratio could be significantly reduced (Figure 5A,F), and SOD2 activity was enhanced (Figure 5G). At the same time, ROS and 8‐OH‐dG were also significantly reduced (Figure 5H–J), and when 3‐TYP pretreatment was given, the effect of ZnG could be significantly reversed. To sum up, the pathway of zinc against oxidative stress is partially achieved by SIRT3 regulating the acetylation level of SOD2 and affecting the activity of SOD2.

### 3.5 Zinc can mediate mitophagy via SIRT3

**FIGURE 6:**
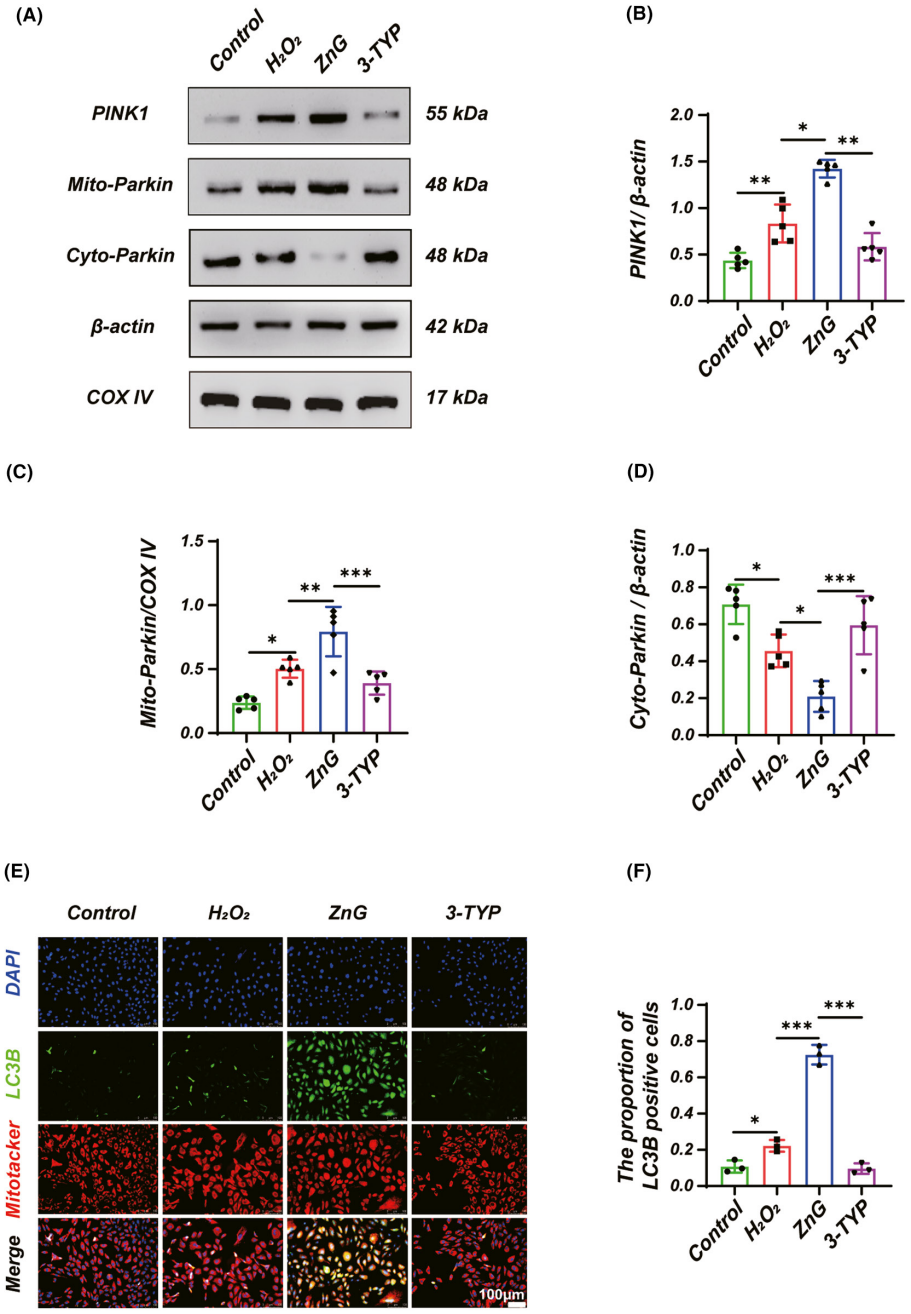
Zinc mediates mitophagy via SIRT3 in VSC4.1 neurons. (A) Western blot and (B) statistical results of western blots of PINK1 (*n* = 5, one‐way ANOVA followed by Games–Howell post hoc test). (A) Western blot and (C,D) statistical results of western blots of Mito‐Parkin and Cyto‐Parkin (*n* = 5, one‐way ANOVA followed by Bonferroni's post hoc test). (E) Immunofluorescence and (F) statistical analysis of immunofluorescence staining of LC3B from each group (*n* = 3, Scale bar = 100 μm, one‐way ANOVA followed by Bonferroni's post hoc test). The data represent means ± SD, *p < 0.05, **p < 0.01, ***p < 0.001.

It has been shown that SIRT3 is involved in regulating mitophagy. Also, we further explored the effect of SIRT3 on the promotion of mitophagy by ZnG. Western blotting results suggested that when 3‐TYP treatment was given, the OD values of PINK1 and Mito‐Parkin were significantly reduced compared with the ZnG treatment group, and the OD values of Cyto‐Parkin group were significantly increased compared with the ZnG group (Figure 6A–D), suggesting that 3‐TYP reversed the ZnG treatment effect. In addition, we detected LC3B using immunocytofluorescence, and LC3B expression was significantly reduced in the 3‐TYP group (Figure 6E,F). In summary, zinc promotes mitophagy via SIRT3.

### 3.6 Inhibition of SIRT3 reverses the effect of Zinc on Parthanatos

**FIGURE 7:**
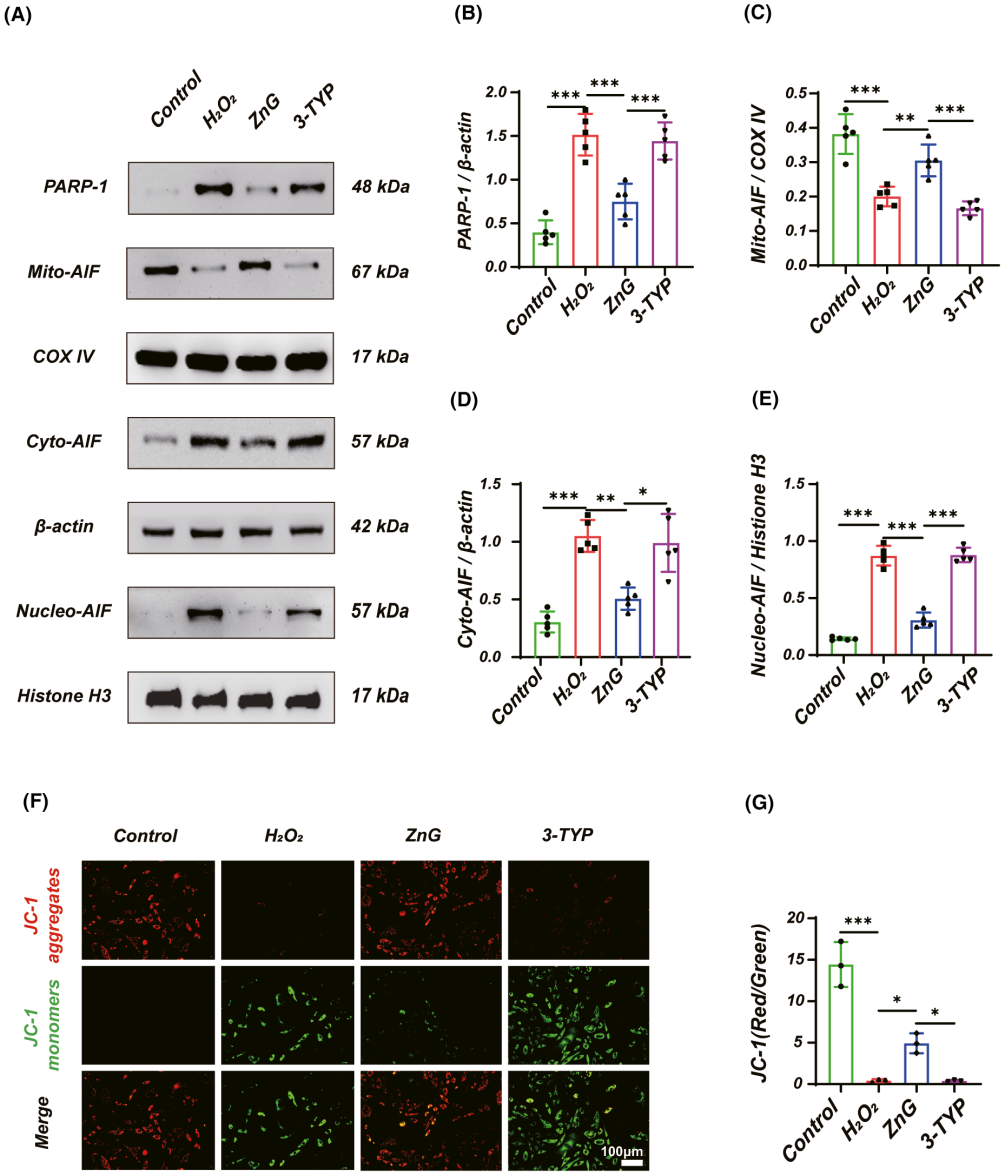
Zinc regulates Parthanatos through SIRT3 in VSC4.1 neurons. (A) Western blot and (B‐E) statistical results of western blots of PARP‐1, Mito‐AIF, Cyto‐AIF, and Nucleo‐AIF (*n* = 5, one‐way ANOVA followed by Bonferroni's post hoc test). (F) JC‐1 staining and (G) statistical analysis of fluorescence (Red/Green) from each group (*n* = 5, Scale bar = 100 μm, one‐way ANOVA followed by Bonferroni's post hoc test). The data represent means ± SD, *p < 0.05, **p < 0.01, ***p < 0.001.

We further clarified the role of SIRT3 in regulating Parthanatos by zinc. In vitro, we assayed parthanatos‐related indicators. Western blotting suggested that PARP‐1 expression was significantly increased in the 3‐TYP group compared with the ZnG group, implying that 3‐TYP could reverse the therapeutic effect of ZnG (Figure 7A,B). Meanwhile, we also detected the change of AIF in vitro using Western blotting, and the results showed that the 3‐TYP group could reverse the nuclear translocation of AIF in the ZnG group (Figure 7A,C–E). In addition, we used a JC‐1 fluorescent probe to detect the mitochondrial membrane potential, and the 3‐TYP group showed a significant increase compared to the ZnG JC‐1 monomer, suggesting that 3‐TYP reverses the therapeutic effect of ZnG (Figure 7F,G). In summary, SIRT3 is involved in the regulation of Parthanatos by zinc.

### 3.7 Inhibition of SIRT3 reverses the effect of zinc in promoting recovery of function after spinal cord injury

**FIGURE 8:**
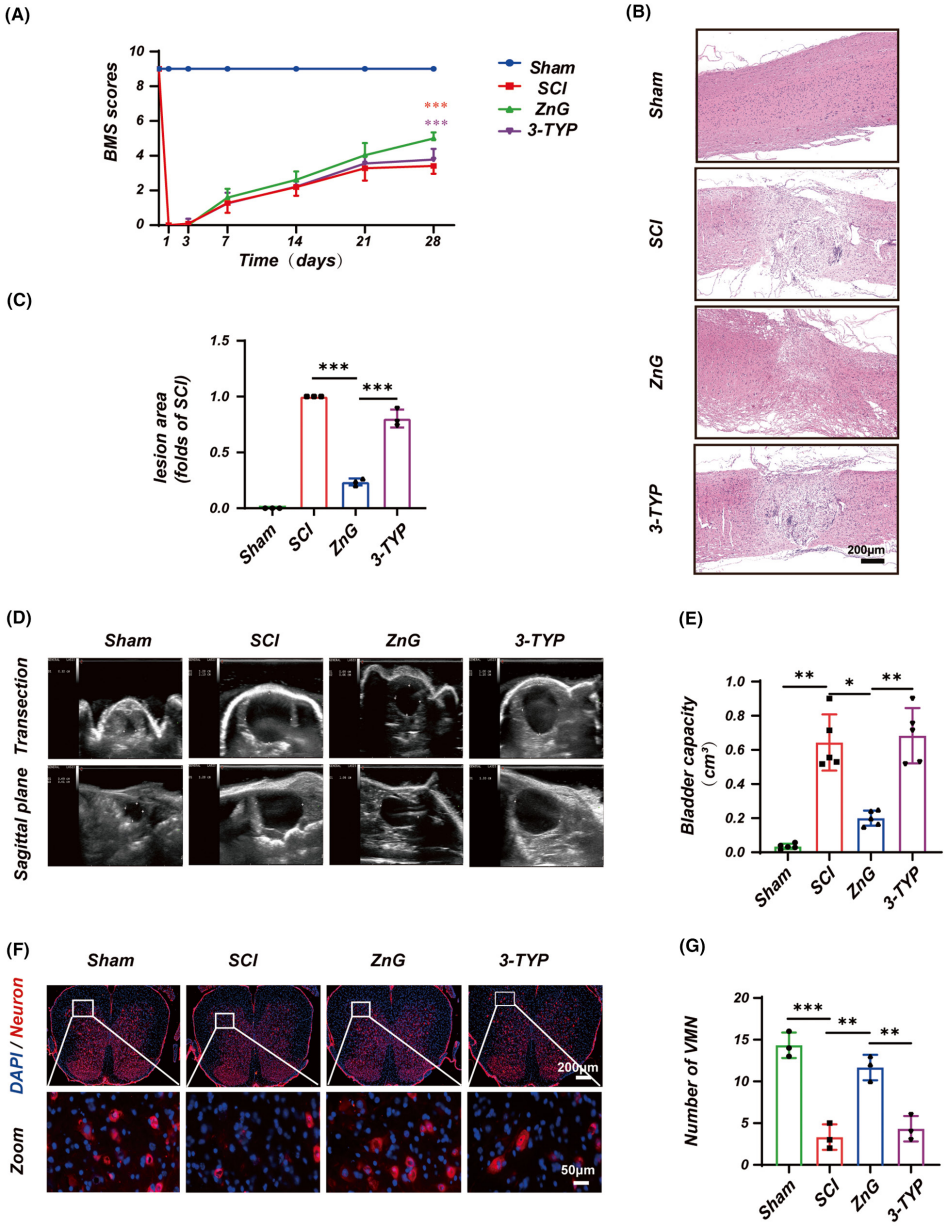
Zinc can promote the recovery of motor function of SCI mice via SIRT3. (A) Statistical results of BMS scores (*n* = 10, non‐parametric Kruskal‐Wallis H test followed by Bonferroni correction). (B) The HE staining and (C) statistical results in 28 days (*n* = 3, Scale bar = 200 μm, the data represent means ± SD, one‐way ANOVA followed by Bonferroni's post hoc test). (D) Ultrasound detects and (E) statistical results of urinary retention at 28 days (*n* = 5, the data represent means ± SD, one‐way ANOVA followed by Games–Howell post hoc test). (F) Immunofluorescence and (G) statistical analysis of the number of neurons in the ventral horn of the spinal cord (Scale bar = 200 μm and 50 μm, the data represent means ± SD, one‐way ANOVA followed by Bonferroni's post hoc test). *p < 0.05, **p < 0.01, ***p < 0.001.

BMS scores were used to assess zinc's effect on motor function recovery after SCI. The results showed that mice in the ZnG group scored better than the SCI group at 28 and 35 days after SCI, and the 3‐TYP group on postoperative days 28 and 35 were significantly lower than those of the ZnG group (Figure 8A). We performed HE staining on the spinal cord of each group 28 days after SCI to observe the changes in the injury site. The results showed that the area of spinal cord injury in the ZnG group was significantly reduced compared with the SCI group and more prominent in the 3‐TYP group than in the ZnG group (Figure 8B,C). The degree of recovery of bladder function after SCI indirectly reflected the recovery of the spinal cord. Therefore, we assessed the bladder of mice 28 days after SCI using ultrasound imaging urinary retention. The results showed that urinary retention was more advanced in the ZnG group mice than in the SCI group and decreased in the 3‐TYP‐treated group compared with the ZnG treatment (Figure 8D,E). In addition, we also performed immunofluorescence staining of spinal cord tissue. The ZnG group restored the number of motor neurons in the anterior horn of the spinal cord compared with the SCI group. The 3‐TYP group could significantly inhibit the number of motor neurons in the anterior horn of the spinal cord compared with ZnG (Figure 8F,G). To sum up, inhibition of SIRT3 reversed the effect of zinc on functional recovery after spinal cord injury.

## 4 DISCUSSION

**FIGURE 9:**
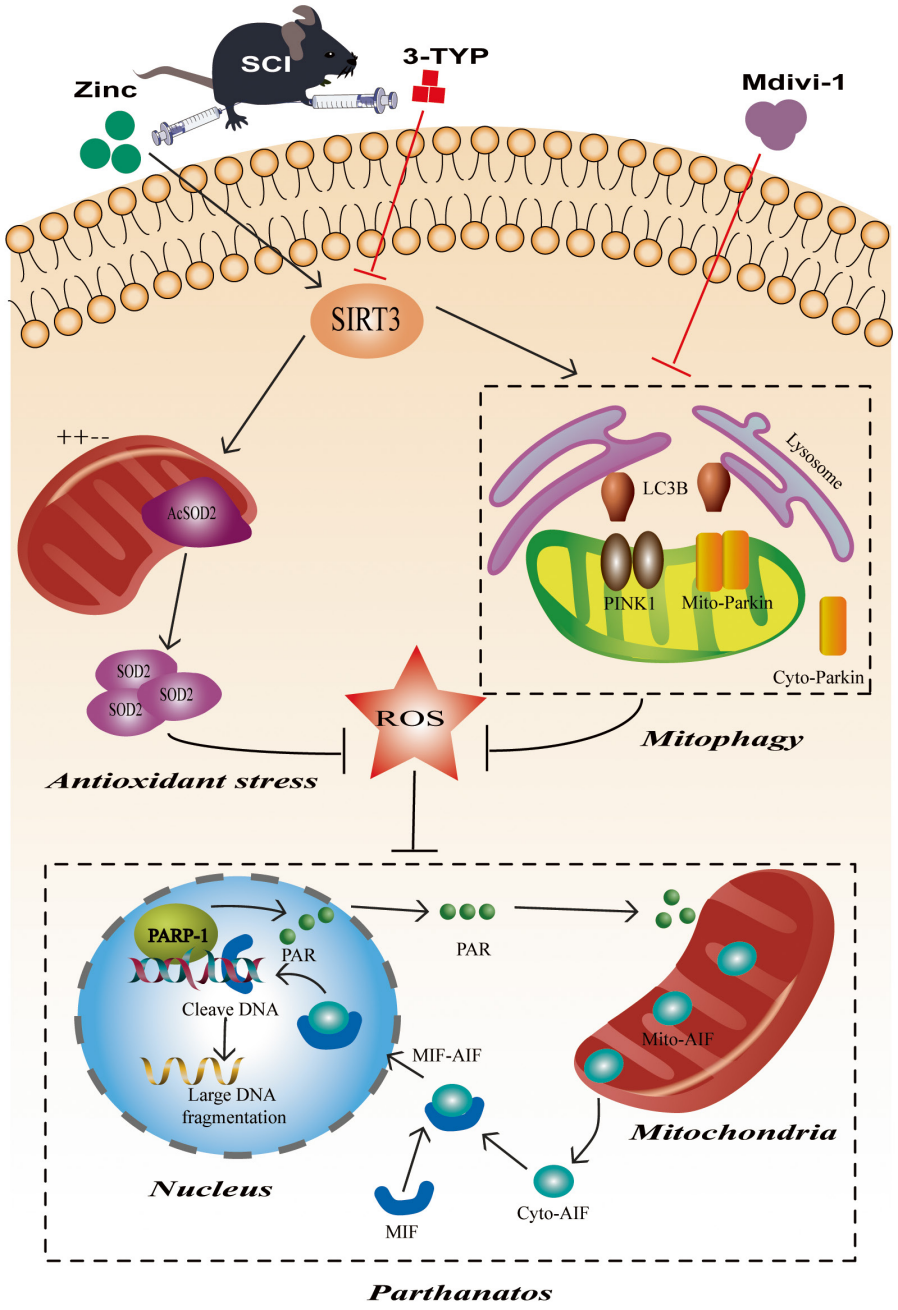
The scheme of zinc regulating Parthanatos after SCI. Zinc defends against Parthanatos and promotes functional recovery after spinal cord injury through SIRT3‐mediated anti‐oxidative stress and mitophagy.

Traumatic spinal cord injury is the most common type of spinal cord injury, resulting in immediate or delayed paraplegia in approximately 11%–40% of patients, which causes long‐term morbidity and high medical costs for patients worldwide.^40^ Zinc is essential for life activities, and studies have shown that zinc is involved in oxidative stress, inflammatory responses, and immune regulation.^23^, ^29^, ^30^ Our previous studies confirmed that zinc protects mitochondria, protects neurons, and promotes spinal cord recovery.^31^, ^32^ This study presents new preclinical evidence that zinc may contribute to functional recovery after spinal cord injury. Mechanistically, the therapeutic effect of zinc may be the inhibition of Parthanatos by scavenging ROS through the direct pathway of SIRT3‐mediated antioxidant stress and indirectly inhibiting ROS accumulation through the mitophagy path (Figure 9).

Oxidative stress after spinal cord injury generates large amounts of ROS, disrupting the body's oxidative/antioxidant system, and oxidative damage to proteins, lipids, and nucleic acids is the leading cause of multiple cell deaths.^4^ Parthanatos is a programmed death dependent on poly ADP‐ribose polymerase‐1 (PARP‐1), in which the accumulation of ROS plays a vital role in activating Parthanatos.^7^ Oxidative DNA damage is the leading cause of PARP‐1 activation, which further induces cascade events such as PAR synthesis and release, mitochondrial depolarization, reduction of mitochondrial membrane potential, and nuclear translocation of AIF.^7^ Therefore, anti‐oxidative stress is an important target for treating spinal cord injuries. Our study showed that SOD2 activity was significantly enhanced after zinc treatment, which can directly scavenge ROS and protect mitochondria; however, it is interesting that AcSOD2/SOD2 ratio was reduced considerably after zinc treatment, suggesting that zinc is enhancing SOD2 activity to scavenge ROS through the deacetylation pathway. While SIRT3, a deacetylase, has been shown to target SOD2.^41^, ^42^ Meanwhile, we examined the changes in SIRT3 after the administration of zinc treatment and found that SIRT3 was significantly reduced. When we administered the SIRT3 inhibitor 3‐TYP, we significantly reversed the therapeutic effect of zinc, leading to an increase in ROS and activation of Parthanatos. We conclude that zinc ions are directly scavenging ROS, thus regulating Parthanatos through the SIRT3‐targeted SOD2 pathway.

Mitochondria are the primary site of ROS production, which can further damage mitochondria, creating a vicious cycle that increases oxidative DNA damage and induces cell death.^13^ Therefore, removing damaged mitochondria is critical for cell survival and controlling the source of mitochondrial damage. The selective degradation of damaged mitochondria by autophagy is known as mitophagy, which is extremely important for maintaining mitochondrial homeostasis.^17^ Studies have shown that mitophagy can indirectly reduce ROS accumulation and prevent further neuronal damage.^43^

Thus, we explored the hypothesis of whether enhanced mitophagy after zinc treatment is the underlying mechanism of zinc inhibition of Parthanatos after spinal cord injury. The results showed that zinc treatment significantly increased LC3B, PINK1, and Mito‐Parkin levels after spinal cord injury, suggesting an upregulation of mitophagy accompanied by a decrease in ROS, 8‐OHdG, and Parthanatos protein expression. When Midivi‐1, a mitophagy‐specific inhibitor, was administered, the expression of ROS, 8‐OHdG and Parthanatos‐related factors increased significantly, confirming that zinc reduces ROS accumulation by enhancing mitophagy to minimize the occurrence of Parthanatos. Interestingly, when we administered the SIRT3‐specific inhibitor 3‐TYP, we found a decrease in the expression of mitophagy‐related proteins, which confirms the involvement of SIRT3 in regulating mitophagy.

The current study has several limitations. For example, whether the regulation of mitophagy by SIRT3 is multifactorial and the specific mechanism remains further explored. The feedback regulation caused by ROS changes is also one of the factors that cannot be ignored. Secondly, oxidative damage of proteins and nucleic acids generated by oxidative stress can lead to the mutual occurrence or promotion of multiple programmed deaths. The specific mechanisms also deserve further investigation. Besides acting on neurons, the relevant roles between glial cells and neurons remain unclear, and other studies are necessary.

## 5 CONCLUSION

In summary, we demonstrate that zinc protects SCI mice by regulating Parthanatos. The protective mechanism is closely related to mitochondria, and the regulation of Parthanatos of zinc is multifaceted. On the one hand, zinc eliminates ROS through SIRT3 deacetylation targeting SOD2 to alleviate Parthanatos; on the other hand, zinc indirectly eliminates ROS through SIRT3‐mediated promotion of mitophagy to alleviate Parthanatos. Therefore, zinc therapy is a promising and effective treatment strategy for SCI.

## AUTHOR CONTRIBUTIONS

D.J. completed the conception and design of the whole experiment. D.J., X.Y., G.M., H.H., and S.W. were involved in behavioral scoring and sample preparation D.J., C.X., and H.D. were involved with cell cultures. D.J. finished statistical analysis and manuscript preparation. Prof X.M. completed the final review and submitted the manuscript. All authors provided important intellectual content to the manuscript and approved its publication.

## CONFLICT OF INTEREST STATEMENT

The authors declare that they have no competing interests.

## Supporting information


Appendix S1:

## Data Availability

The raw data of experiments used to support the findings of this study are available from the corresponding author upon request.

## References

[1] Sekhon LH , Fehlings MG . Epidemiology, demographics, and pathophysiology of acute spinal cord injury. Spine (Phila Pa 1976). 2001;26:S2‐S12. 11805601 10.1097/00007632-200112151-00002

[2] GBD 2016 Traumatic Brain Injury and Spinal Cord Injury Collaborators . Global, regional, and national burden of traumatic brain injury and spinal cord injury, 1990–2016: a systematic analysis for the global burden of disease study 2016. Lancet Neurol. 2019;18:56‐87. 30497965 10.1016/S1474-4422(18)30415-0 PMC6291456

[3] Alizadeh A , Dyck SM , Karimi‐Abdolrezaee S . Traumatic spinal cord injury: an overview of pathophysiology, models and acute injury mechanisms. Front Neurol. 2019;10:282. 30967837 10.3389/fneur.2019.00282 PMC6439316

[4] Anjum A , Yazid MD , Fauzi Daud M , et al. Spinal cord injury: pathophysiology, multimolecular interactions, and underlying recovery mechanisms. Int J Mol sci. 2020;21:7533. 33066029 10.3390/ijms21207533 PMC7589539

[5] Dimitrijevic MR , Danner SM , Mayr W . Neurocontrol of movement in humans with spinal cord injury. Artif Organs. 2015;39:823‐833. 26471132 10.1111/aor.12614

[6] Harraz MM , Dawson TM , Dawson VL . Advances in neuronal cell death 2007. Stroke. 2008;39:286‐288. 18187674 10.1161/STROKEAHA.107.511857

[7] Galluzzi L , Vitale I , Aaronson SA , et al. Molecular mechanisms of cell death: recommendations of the nomenclature committee on cell death 2018. Cell Death Differ. 2018;25:486‐541. 29362479 10.1038/s41418-017-0012-4 PMC5864239

[8] Wang Y , An R , Umanah GK , et al. A nuclease that mediates cell death induced by DNA damage and poly(ADP‐ribose) polymerase‐1. Science. 2016;354:aad6872. 27846469 10.1126/science.aad6872 PMC5134926

[9] Andrabi SA , Kim NS , Yu SW , et al. Poly(ADP‐ribose) (PAR) polymer is a death signal. Proc Natl Acad sci USA. 2006;103:18308‐18313. 17116882 10.1073/pnas.0606526103 PMC1838747

[10] Paddock MN , Buelow BD , Takeda S , Scharenberg AM . Correction: the BRCT domain of PARP‐1 is required for immunoglobulin gene conversion. PLoS Biol. 2018;16:e1002621. 29494577 10.1371/journal.pbio.1002621 PMC5832193

[11] Baek SH , Bae ON , Kim EK , Yu SW . Induction of mitochondrial dysfunction by poly(ADP‐ribose) polymer: implication for neuronal cell death. Mol Cells. 2013;36:258‐266. 23996529 10.1007/s10059-013-0172-0 PMC3887971

[12] Fatokun AA , Dawson VL , Dawson TM . Parthanatos: mitochondrial‐linked mechanisms and therapeutic opportunities. Br J Pharmacol. 2014;171:2000‐2016. 24684389 10.1111/bph.12416 PMC3976618

[13] Bock FJ , Tait S . Mitochondria as multifaceted regulators of cell death. Nat Rev Mol Cell Biol. 2020;21:85‐100. 31636403 10.1038/s41580-019-0173-8

[14] Golpich M , Amini E , Mohamed Z , Azman Ali R , Mohamed Ibrahim N , Ahmadiani A . Mitochondrial dysfunction and biogenesis in neurodegenerative diseases: pathogenesis and treatment. CNS Neurosci Ther. 2017;23:5‐22. 27873462 10.1111/cns.12655 PMC6492703

[15] Jha RM , Kochanek PM , Simard JM . Pathophysiology and treatment of cerebral edema in traumatic brain injury. Neuropharmacology. 2019;145:230‐246. 30086289 10.1016/j.neuropharm.2018.08.004 PMC6309515

[16] Chen X , Cui J , Zhai X , et al. Inhalation of hydrogen of different concentrations ameliorates spinal cord injury in mice by protecting spinal cord neurons from apoptosis, oxidative injury and mitochondrial structure damages. Cell Physiol Biochem. 2018;47:176‐190. 29763919 10.1159/000489764

[17] Li Q , Gao S , Kang Z , et al. Rapamycin enhances mitophagy and attenuates apoptosis after spinal ischemia‐reperfusion injury. Front Neurosci. 2018;12:865. 30559639 10.3389/fnins.2018.00865 PMC6286985

[18] Lin Q , Li S , Jiang N , et al. PINK1‐parkin pathway of mitophagy protects against contrast‐induced acute kidney injury via decreasing mitochondrial ROS and NLRP3 inflammasome activation. Redox Biol. 2019;26:101254. 31229841 10.1016/j.redox.2019.101254 PMC6597739

[19] Chu CT . Mechanisms of selective autophagy and mitophagy: implications for neurodegenerative diseases. Neurobiol Dis. 2019;122:23‐34. 30030024 10.1016/j.nbd.2018.07.015 PMC6396690

[20] Singh CK , Chhabra G , Ndiaye MA , Garcia‐Peterson LM , Mack NJ , Ahmad N . The role of Sirtuins in antioxidant and redox signaling. Antioxid Redox Signal. 2018;28:643‐661. 28891317 10.1089/ars.2017.7290 PMC5824489

[21] Bell EL , Guarente L . The SirT3 divining rod points to oxidative stress. Mol Cell. 2011;42:561‐568. 21658599 10.1016/j.molcel.2011.05.008 PMC3526939

[22] Chasapis CT , Ntoupa PA , Spiliopoulou CA , Stefanidou ME . Recent aspects of the effects of zinc on human health. Arch Toxicol. 2020;94:1443‐1460. 32394086 10.1007/s00204-020-02702-9

[23] Kitamura H , Morikawa H , Kamon H , et al. Toll‐like receptor‐mediated regulation of zinc homeostasis influences dendritic cell function. Nat Immunol. 2006;7:971‐977. 16892068 10.1038/ni1373

[24] Andreini C , Banci L , Bertini I , Rosato A . Counting the zinc‐proteins encoded in the human genome. J Proteome Res. 2006;5:196‐201. 16396512 10.1021/pr050361j

[25] Ghinis‐Hozumi Y , González‐Gallardo A , González‐Dávalos L , et al. Bovine sirtuins: initial characterization and expression of sirtuins 1 and 3 in liver, muscle, and adipose tissue. J Anim sci. 2011;89:2529‐2536. 21421831 10.2527/jas.2010-3476

[26] Marchler‐Bauer A , Anderson JB , Chitsaz F , et al. CDD: specific functional annotation with the conserved domain database. Nucleic Acids Res. 2009;37:D205‐D210. 18984618 10.1093/nar/gkn845 PMC2686570

[27] Marchler‐Bauer A , Lu S , Anderson JB , et al. CDD: a conserved domain database for the functional annotation of proteins. Nucleic Acids Res. 2011;39:D225‐D229. 21109532 10.1093/nar/gkq1189 PMC3013737

[28] Maret W . Zinc biochemistry: from a single zinc enzyme to a key element of life. Adv Nutr. 2013;4:82‐91. 23319127 10.3945/an.112.003038 PMC3648744

[29] Pae M , Meydani SN , Wu D . The role of nutrition in enhancing immunity in aging. Aging Dis. 2012;3:91‐129. 22500273 PMC3320807

[30] Prasad AS . Zinc: role in immunity, oxidative stress and chronic inflammation. Curr Opin Clin Nutr Metab Care. 2009;12:646‐652. 19710611 10.1097/MCO.0b013e3283312956

[31] Hu H , Xia N , Lin J , et al. Zinc regulates glucose metabolism of the spinal cord and neurons and promotes functional recovery after spinal cord injury through the AMPK signaling pathway. Oxid Med Cell Longev. 2021;2021:4331625. 34373765 10.1155/2021/4331625 PMC8349299

[32] Li D , Tian H , Li X , et al. Zinc promotes functional recovery after spinal cord injury by activating Nrf2/HO‐1 defense pathway and inhibiting inflammation of NLRP3 in nerve cells. Life sci. 2020;245:117351. 31981629 10.1016/j.lfs.2020.117351

[33] Zhai M , Li B , Duan W , et al. Melatonin ameliorates myocardial ischemia reperfusion injury through SIRT3‐dependent regulation of oxidative stress and apoptosis. J Pineal Res. 2017;63:e12419. 10.1111/jpi.12419 28500761

[34] Basso DM , Fisher LC , Anderson AJ , Jakeman LB , McTigue DM , Popovich PG . Basso mouse scale for locomotion detects differences in recovery after spinal cord injury in five common mouse strains. J Neurotrauma. 2006;23:635‐659. 16689667 10.1089/neu.2006.23.635

[35] Keirstead HS , Fedulov V , Cloutier F , Steward O , Duel BP . A noninvasive ultrasonographic method to evaluate bladder function recovery in spinal cord injured rats. Exp Neurol. 2005;194:120‐127. 15899249 10.1016/j.expneurol.2005.01.027

[36] Xu H , Gan C , Gao Z , et al. Caffeine targets SIRT3 to enhance SOD2 activity in mitochondria. Front Cell Dev Biol. 2020;8:822. 33015038 10.3389/fcell.2020.00822 PMC7493682

[37] Lin JQ , Tian H , Zhao XG , et al. Zinc provides neuroprotection by regulating NLRP3 inflammasome through autophagy and ubiquitination in a spinal contusion injury model. CNS Neurosci Ther. 2021;27:413‐425. 33034415 10.1111/cns.13460 PMC7941232

[38] Thangaraj A , Periyasamy P , Liao K , et al. HIV‐1 TAT‐mediated microglial activation: role of mitochondrial dysfunction and defective mitophagy. Autophagy. 2018;14:1596‐1619. 29966509 10.1080/15548627.2018.1476810 PMC6135576

[39] Ge MH , Tian H , Mao L , et al. Zinc attenuates ferroptosis and promotes functional recovery in contusion spinal cord injury by activating Nrf2/GPX4 defense pathway. CNS Neurosci Ther. 2021;27:1023‐1040. 33951302 10.1111/cns.13657 PMC8339532

[40] Hutson TH , Di Giovanni S . The translational landscape in spinal cord injury: focus on neuroplasticity and regeneration. Nat Rev Neurol. 2019;15:732‐745. 31728042 10.1038/s41582-019-0280-3

[41] Gao J , Feng Z , Wang X , et al. SIRT3/SOD2 maintains osteoblast differentiation and bone formation by regulating mitochondrial stress. Cell Death Differ. 2018;25:229‐240. 28914882 10.1038/cdd.2017.144 PMC5762839

[42] Ma LL , Kong FJ , Dong Z , et al. Hypertrophic preconditioning attenuates myocardial Ischaemia‐reperfusion injury by modulating SIRT3‐SOD2‐mROS‐dependent autophagy. Cell Prolif. 2021;54:e13051. 33973685 10.1111/cpr.13051 PMC8249780

[43] Wu C , Chen H , Zhuang R , et al. Betulinic acid inhibits pyroptosis in spinal cord injury by augmenting autophagy via the AMPK‐mTOR‐TFEB signaling pathway. Int J Biol sci. 2021;17:1138‐1152. 33867836 10.7150/ijbs.57825 PMC8040310

