# Supplementary material for: Zinc defends against Parthanatos and promotes functional recovery after spinal cord injury through SIRT3‐mediated anti‐oxidative stress and mitophagy
Source: CNS Neurosci Ther. 2023 Apr 17;29(10):2857–72. doi: 10.1111/cns.14222 (PMC10493669; doi:10.1111/cns.14222)

Full unedited blot for Figure 1A PARP-1

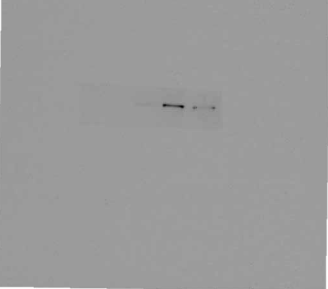

Full unedited blot for Figure 1A β-actin

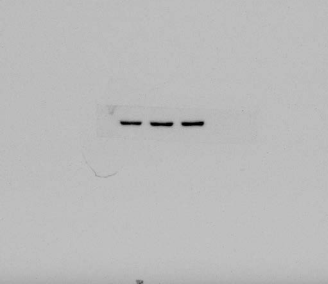

Full unedited blot for Figure 1F Mito-AIF

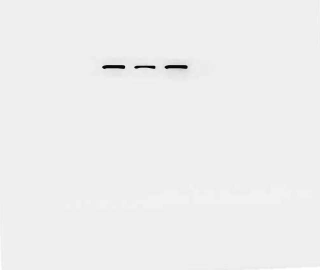

Full unedited blot for Figure 1F COX IV

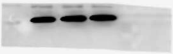

Full unedited blot for Figure 1H Cyto-AIF

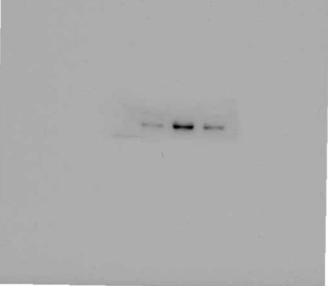

Full unedited blot for Figure 1H β-actin

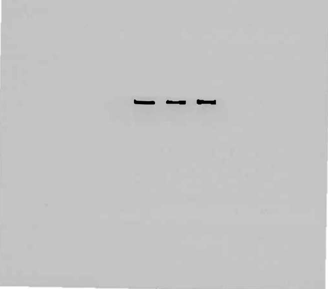

Full unedited blot for Figure 1J Nucleo-AIF

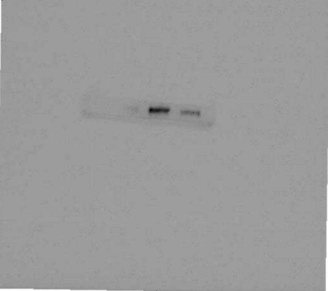

Full unedited blot for Figure 1J Histone H3

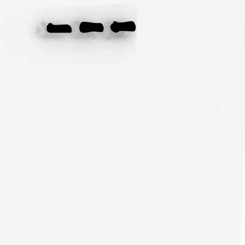

Full unedited blot for Figure 2B LC3A/B

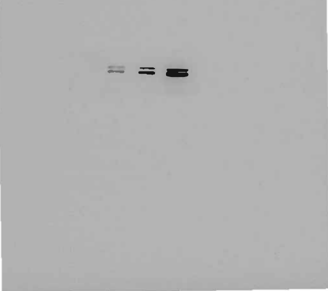

Full unedited blot for Figure 2B β-actin

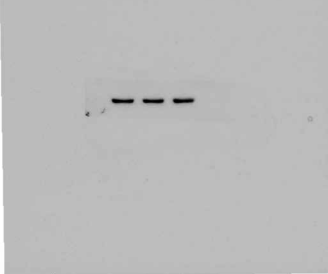

Full unedited blot for Figure 2F PINK1

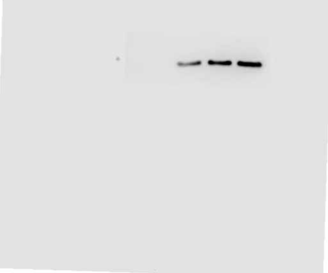

Full unedited blot for Figure 2F β-actin

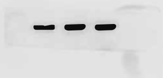

Full unedited blot for Figure 2H Mito-Parkin

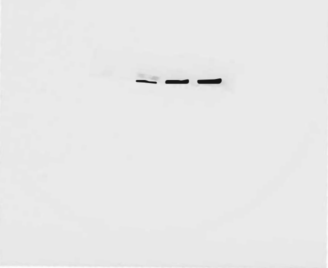

Full unedited blot for Figure 2H COX IV

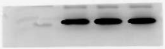

Full unedited blot for Figure 2J Cyto-Parkin

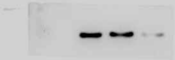

Full unedited blot for Figure 2J β-actin

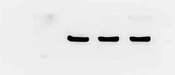

Full unedited blot for Figure 3E PARP-1

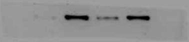

Full unedited blot for Figure 3E β-actin

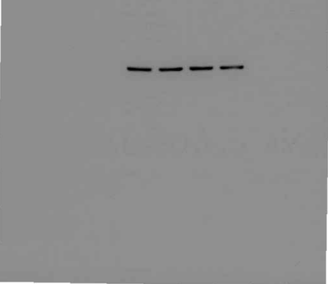

Full unedited blot for Figure 4B Mito-AIF

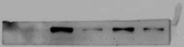

Full unedited blot for Figure 4B COX IV

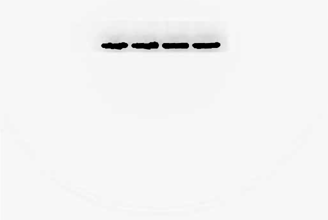

Full unedited blot for Figure 4D Cyto-AIF

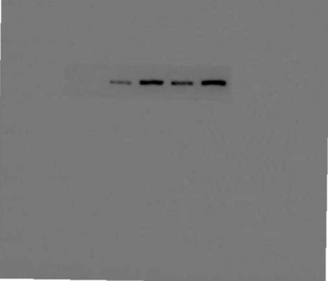

Full unedited blot for Figure 4D β-actin

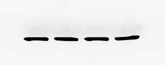

Full unedited blot for Figure 4F Nucleo-AIF

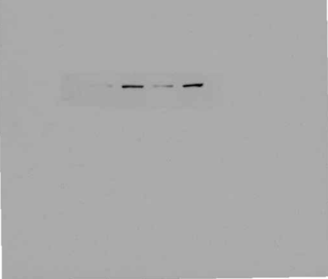

Full unedited blot for Figure 4F Histone H3

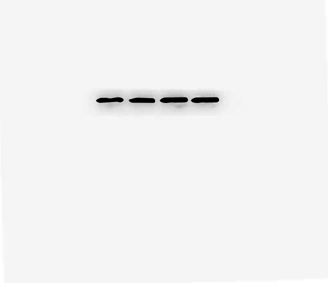

Full unedited blot for Figure 5A SIRT3

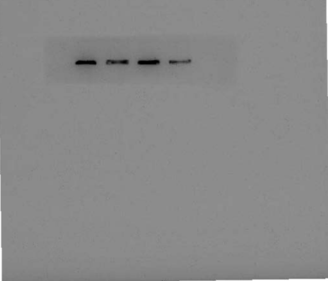

Full unedited blot for Figure 5A AcSOD2

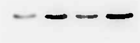

Full unedited blot for Figure 5A SOD2

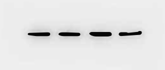

Full unedited blot for Figure 5A β-actin

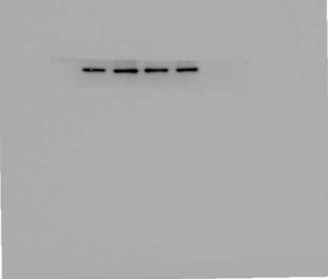

Full unedited blot for Figure 6A PINK1

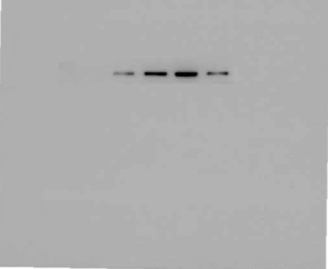

Full unedited blot for Figure 6A Mito-Parkin

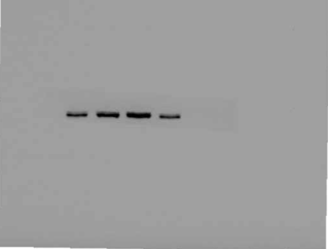

Full unedited blot for Figure 6A COX IV

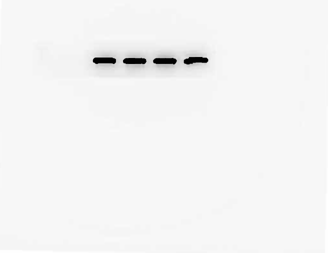

Full unedited blot for Figure 6A Cyto-Parkin

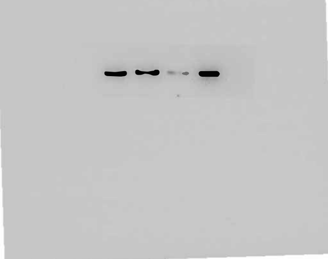

Full unedited blot for Figure 6A β-actin

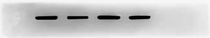

Full unedited blot for Figure 7A PARP-1

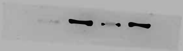

Full unedited blot for Figure 7A Mito-AIF

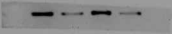

Full unedited blot for Figure 7A COX IV

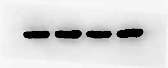

Full unedited blot for Figure 7A Cyto-AIF

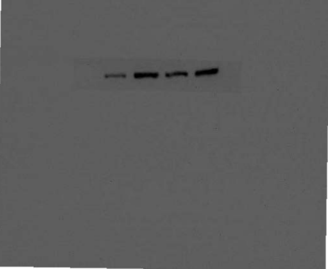

Full unedited blot for Figure 7A β-actin

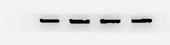

Full unedited blot for Figure 7A Cyto-AIF

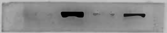

Full unedited blot for Figure 7A Histone H3

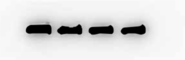

Supplement: Supplementary file 1 — Appendix S1: [file CNS-29-2857-s001.zip › CNS_14222_Supplemental Files.pdf]
