# Supplementary figures and images for: Zinc defends against Parthanatos and promotes functional recovery after spinal cord injury through SIRT3‐mediated anti‐oxidative stress and mitophagy
Source: CNS Neurosci Ther. 2023 Apr 17;29(10):2857–72. doi: 10.1111/cns.14222 (PMC10493669; doi:10.1111/cns.14222)

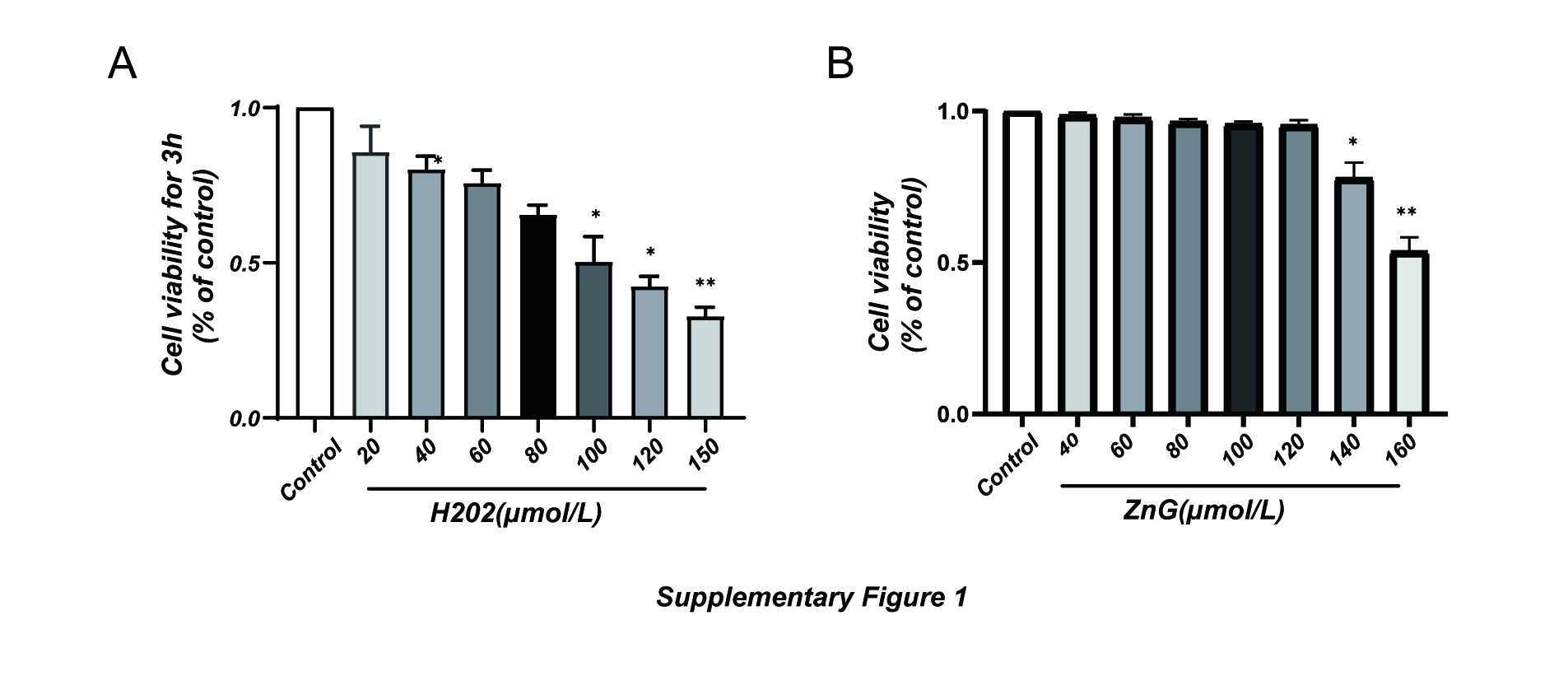

Supplement: Supplementary file 1 — Appendix S1: [file CNS-29-2857-s001.zip › CNS_14222_Fig.S1.tif]
